# Species composition and relative abundance of the genus *Culicoides* (Diptera: Ceratopogonidae) in Romania

**DOI:** 10.1186/s13071-020-04247-3

**Published:** 2020-08-03

**Authors:** Doru Hristescu, Florica Bărbuceanu, Lenuța Dascălu, Cristina Nițescu, Maria Goffredo, Adriana Santilli, Michela Quaglia, Thomas Balenghien, Gabriel Predoi

**Affiliations:** 1 Institute for Diagnosis and Animal Health, Bucharest, Romania; 2 Faculty of Veterinary Medicine, Bucharest, Romania; 3 Istituto Zooprofilattico Sperimentale dell’Abruzzo e del Molise ‘G. Caporale’, Teramo, Italy; 4 ASTRE, University of Montpellier, Cirad, INRA, Montpellier, France; 5 Cirad, UMR ASTRE, 10101 Rabat, Morocco; 6 Unité Parasitologie et maladies parasitaires, Institut Agronomique et Vétérinaire Hassan II, 10100 Rabat, Morocco

**Keywords:** Romania, *Culicoides*, Entomological surveillance, Bluetongue

## Abstract

**Background:**

*Culicoides* biting midges are vectors involved in the biological transmission cycle of important animal diseases such as bluetongue and African horse sickness. In Romania, the first outbreaks of bluetongue were reported in 2014, leading to increased activities within the existing entomological surveillance network. The main goals of the surveillance activities were the establishment of the vector free period in relation to animal trade and the identification of *Culicoides* species involved in the transmission of the pathogen. This study was conducted on the composition and relative abundance of the species belonging to the genus *Culicoides* (Diptera: Ceratopogonidae) in certain regions of Romania and provided the opportunity to update the existing checklist of *Culicoides* species of this country.

**Methods:**

The study was conducted in 33 of the 42 administrative units (counties), including a total of 659 catches, in 102 locations. The collections were carried out with UV blacklight suction traps (OVI type). The collected insects were preserved in 70% ethanol. Morphological insect identification was carried out using a stereomicroscope, according to established identification keys. In ten localities the relative abundance of the cryptic species of the Obsoletus complex was determined by multiplex PCR assay based on the ITS2 segment. The identification of the *Culicoides chiopterus* (Meigen) species by morphological examination was confirmed by PCR assay based on the ITS1 segment.

**Results:**

Eleven species were identified using morphological and PCR tools. The rest of the individuals were separated into five taxa. The species of the Obsoletus complex (grouping *Culicoides obsoletus* (Meigen) and *Culicoides scoticus* Downes & Kettle) were the most abundant, accounting for 59% of the total number of captured *Culicoides* spp. Three of the identified species are mentioned, according to our knowledge, for the first time in Romania: *Culicoides newsteadi* Austen, *Culicoides flavipulicaris* Dzhafarov and *Culicoides bysta* Sarvašová, Kočisová, Candolfi & Mathieu.

**Conclusions:**

Our study demonstrates that the *Culicoides* species most commonly cited as being involved in the transmission of arboviruses in Europe (i.e. bluetongue and Schmallenberg viruses) make up a high proportion of adult *Culicoides* trapped in Romania. 
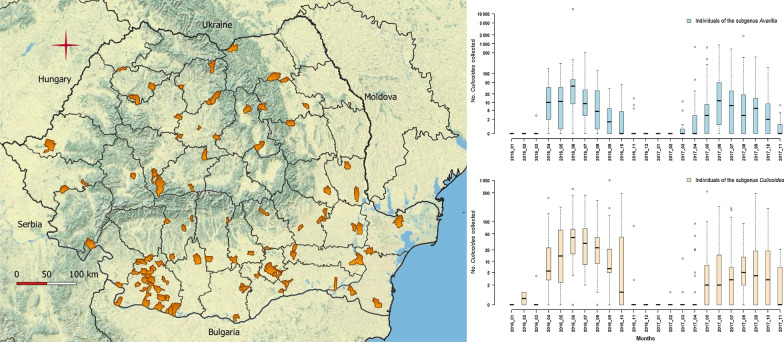

## Background

In the last two decades the emergence and spread at European level of arthropod borne diseases such as bluetongue or Schmallenberg disease, highlighted the importance of *Culicoides* biting midges as vectors involved in the biological transmission cycle [1–4]. When bluetongue (BT) disease started to spread into mainland Europe at the beginning of the 2000s, an entomological surveillance network for *Culicoides* biting midges was set up in Romania in 2003, as part of an active and passive surveillance programme, aimed at early detection of bluetongue virus in the local population of domestic ruminants. As the disease generated significant economic losses [5], the main goal of the entomological surveillance programme was to establish the start and the end of the vector-free period. Indeed, the European regulations allow a country to reduce the restrictions on animal movements during the winter period of low activity of *Culicoides* spp.. The national status of being free of bluetongue disease changed for Romania in August 2014 when the first outbreaks were described in the Buzău county, caused by serotype 4 of BTV (BTV-4) [6–9]. This emergence was related to the massive BTV-4 epizootic which started in the Balkan Peninsula in May 2014. Following the declaration of the first outbreaks, the disease spread rapidly in most regions, with a total of 1885 outbreaks in 658 localities from 35 counties notified in only three months (September to November 2014). In compliance with European legislation, the whole national territory was declared in December 2014 as a restriction zone for BT, with an immediate impact on trade in live ruminants (mainly sheep exports in non-EU countries). In 2015, the clinical impact of BT evolution was significantly different from 2014, with only 30 outbreaks registered in four counties situated in the north-eastern part of the country. In the last few years (2016 to present) no outbreaks related to clinical signs were notified, but the country’s infection status remains as active serological surveillance targeted on sentinel ruminants still provides positive results. In the context of emergence of BT disease in Romania, the importance of entomological surveillance became even higher as the start and the end of the vector free period has an immediate impact on local animal trade. An entomological surveillance was then implemented with this main scope. The *Culicoides* spp. identification was targeted only at the subgenera known to be involved in BTV transmission (*Avaritia* Fox, *Culicoides* Latreille and *Monoculicoides* Khalaf). Consequently, scarce information on species diversity and relative abundance was available following the implementation of the programme.

The last comprehensive *Culicoides* spp. inventory of Romania was published in 2000 [10] reporting 46 species for the Romanian fauna. These species were grouped in eight subgenera: *Trithecoides* Wirth & Hubert (*n* = 1), *Pontoculicoides* Remm (*n* = 2), *Avaritia* Fox (*n* = 5), *Culicoides* Latreille (*n* = 8), *Beltranmyia* Vargas (*n* = 4), *Monoculicoides* Khalaf (*n* = 4), *Sensiculicoides* Schevchenko (*n* = 1) and *Oecacta* Poey (*n* = 21). Since this publication, only a few studies have dealt with the genus *Culicoides*, and reports concerned only few locations with identifications carried out mainly at the group level [11–14]. Collecting biting midges at a few locations of the Danube Delta, a recent publication [15] reported three additional species to the Romanian fauna, i.e. *C. griseidorsum* Kieffer, *C. puncticollis* (Becker) and *C. submaritimus* Dzhafarov, which is considered by some authors as synonym of *C. maritimus* Kieffer, leading to 49 species recorded in Romania.

In this study, we aimed to describe the diversity and relative abundance of *Culicoides* spp. in Romania, using morphological identification keys and molecular assays to examine surveillance samples.

## Methods

**Fig. 1:**
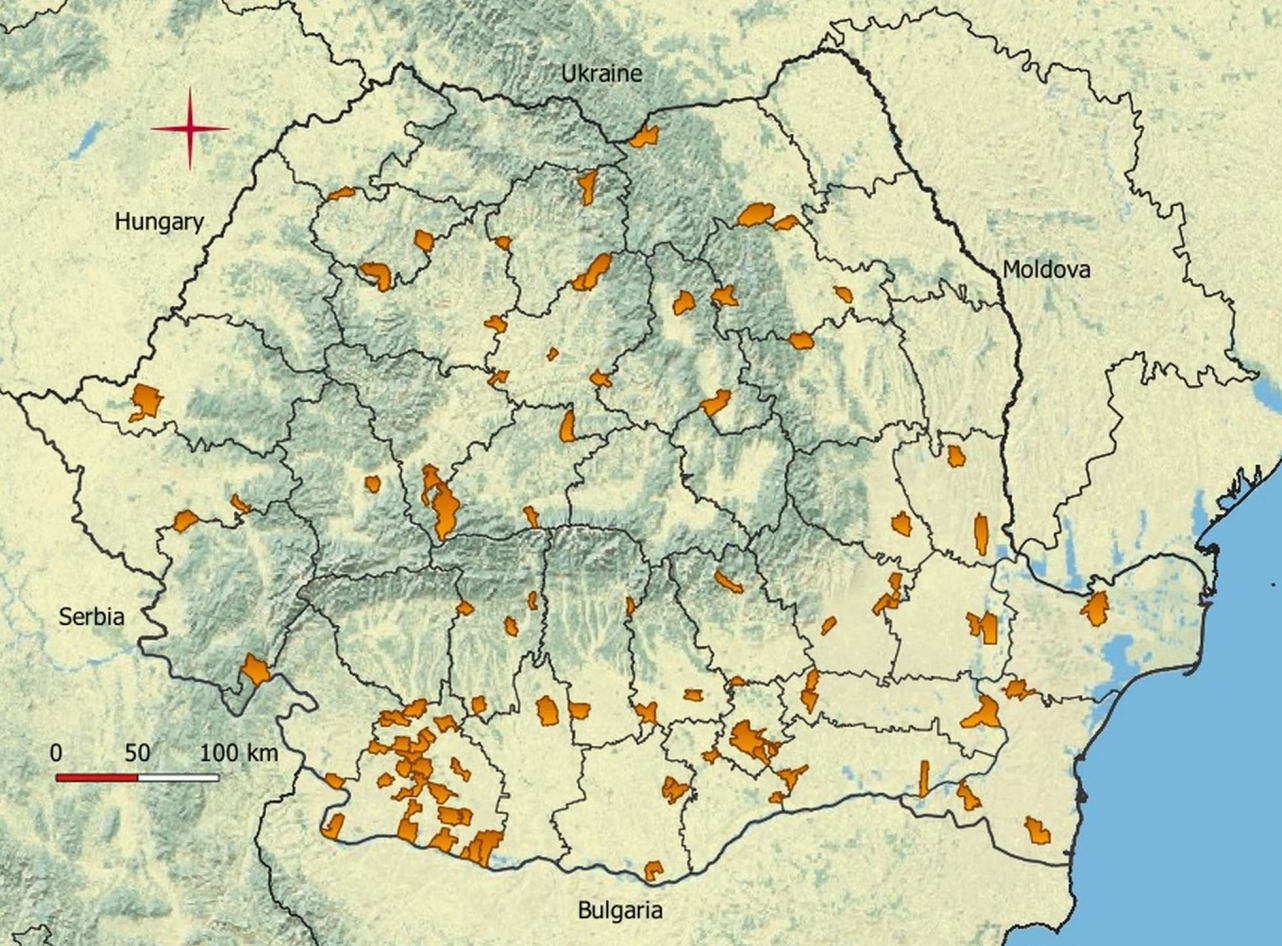
Map of Romania showing trap localities

We gathered entomological samples from the existing national *Culicoides* spp. surveillance network. Samples were collected from 2016 to 2017, in 102 locations distributed in 33 counties (out of a total of 42 administrative units) (Fig. 1). In 28 counties (out of 33) clinical outbreaks of BT were recorded during 2014–2015. Eight locations were commercial farms, of which 6 were cattle farms, 1 semen production unit and 1 mixed farm (cattle, sheep, pigs and horses). The rest of the locations (94) were non-commercial farms (backyards) in which cattle is constantly present, along with sheep and horses (less frequently). The capture locations were selected based on several eligibility criteria: the constant presence of vertebrate species targeted by *Culicoides* spp.; the avoidance of confined spaces; the possibility to install the traps at the height of 1.5–2 m from ground level; and the existence of a power supply. The collections were carried out with UV blacklight suction traps (OVI type), with one trap per site. Collections mostly started in April and ended in October/November to cover the entire *Culicoides* spp. activity period. Additional collections were made taking into account longer or shorter *Culicoides* activity period. The collections were, per county, mostly performed weekly in 2016 (9 counties) and bi-monthly in 2017 (32 counties). The collected insects were preserved in 70% ethanol. Insect identification was carried out using a stereomicroscope, according to the keys provided by Campbell & Pelham-Clinton [16], Delécolle [17], Goffredo and Meiswinkel [18] and Goffredo et al. [19]. Essentially the wing pattern characters were used for species identification; however other parameters, appreciable under the stereomicroscope with a high resolution, were used in addition when needed (i.e. setae on the first abdominal segment, or number and shape of the spermathecae). The *Culicoides* species without a defined wing pattern, including the so called “plain-wing species” [18, 19] were grouped as “other *Culicoides* species”. Due to the difficulties of morphological separation of species within the Obsoletus complex (which includes *C. obsoletus* (*sensu stricto*) (Meigen) and the morphologically close species, *C. scoticus* Downes & Kettle and *C. montanus* Shakirzjanova) the relative abundance of the component species was determined on a limited number of adults. A total of 210 specimens were randomly selected from 10 localities in 10 counties (21 specimens per locality) and individually identified using a multiplex PCR based on the ITS2 segment [19, 20].

The specimens identified morphologically as belonging to *C. chiopterus* (Meigen) were confirmed by a PCR test based on the ITS1 segment [21]. Spatial analysis and maps were generated using QGIS software (version 3.8.2; https://qgis.org).

## Results

A total number of 60,006 *Culicoides* biting midges from 659 catches carried out from 2016 to 2017 were examined. At least 11 species (*n* = 51,200) and 5 taxa (*n* = 2468) belonging to the subgenera *Avaritia*, *Culicoides*, *Monoculicoides*, *Sensiculicoides* and *Beltranmyia* were identified. In addition, 5877 individuals were classified as “other *Culicoides* species”, and 461 individuals could not be identified (broken specimens, with faded wings etc.) (Table 1).

**Table 1:** Total number of *Culicoides* specimens identified in 33 counties in Romania

Subgenus	Species	No. of specimens (%)
*Avaritia*	*C. obsoletus* (Meigen)	35,600 (59.3)
	*C. scoticus* Downes & Kettle	
	*C. dewulfi* Goetghebuer	143 (0.2)
	*C. chiopterus* (Meigen)	6 (0.01)
*Culicoides*	*C. punctatus* (Meigen)	10,080 (16.8)
	*C. newsteadi* Austen	3181 (5.3)
	*C. pulicaris* (Linnaeus)	854 (1.4)
	*C. lupicaris* Downes & Kettle	987 (1.6)
	*C. pulicaris*/*C. lupicaris*	324 (0.5)
	*C. fagineus* Edwards/*C. impunctatus* Goetghebuer	301 (0.5)
	*C. bysta* Sarvašová, Kočisová, Candolfi & Mathieu	131 (0.2)
	*C. flavipulicaris* Dzhafarov	2 (0.003)
*Monoculicoides*	*C. nubeculosus* (Meigen)/*C. riethi* Kieffer/*C. puncticollis* (Becker)	467 (0.8)
	*C. stigma* (Meigen)/*C. parroti* Kieffer	6 (0.01)
*Sensiculicoides*	Festivipennis taxa^a^	1370 (2.2)
*Beltranmyia*	*C. circumscriptus* Kieffer	216 (0.4)
	other *Culicoides* spp.	5877 (9.8)
	*Culicoides* spp.^b^	461 (0.8)

^a^Festivipennis taxa: species such as *C. festivipennis* Kieffer, *C. cataneii* Clastrier, *C. gejgelensis* Dzhafarov

^b^Not identified (i.e. specimens broken, with faded wings) and considered as *Culicoides* spp

The most abundant taxon was the Obsoletus complex, which represented 59.3% (*n* = 35,600) of the total collected midges. Within the subgenus *Avaritia*, *C. dewulfi* Goetghebuer and *C. chiopterus* were also identified (*n* = 143 and *n* = 6, respectively). All the midges identified as *C. chiopterus* were confirmed by PCR. The subgenus *Culicoides* was represented with 26.4% (*n* = 15,860) of the total midges. Within this taxon, 63.6% (n = 10,080) was represented by *C. punctatus* (Meigen), 20.1% (*n* = 3181) were *C. newsteadi* Austen, followed by *C. lupicaris* Downes & Kettle and *C. pulicaris* (Linnaeus) in similar percentages, 6.2% (*n* = 987) and 5.4% (*n* = 854), respectively. Other certain species belonging to this subgenus were identified as *C. bysta* Sarvašová, Kočisová, Candolfi & Mathieu (*n* = 131) and *C. flavipulicaris* Dzhafarov (*n* = 2). Within the subgenus *Beltranmyia* only one species was identified, namely *C. circumscriptus* Kieffer (*n* = 216).

Additionally, other specimens were identified as taxa, including *C. fagineus* Edwards*/C. impunctatus* Goetghebuer (*n* = 301) within the subgenus *Culicoides*, *C. nubeculosus* (Meigen)*/C. riethi* Kieffer*/C. puncticollis* (Becker) (*n* = 467) and *C. stigma* (Meigen)*/C. parroti* Kieffer (*n* = 6) within the subgenus *Monoculicoides*, and species of the Festivipennis taxa (*n* = 1370) within the subgenus *Sensiculicoides*.

### *Culicoides imicola* was never found in this study

**Fig. 2:**
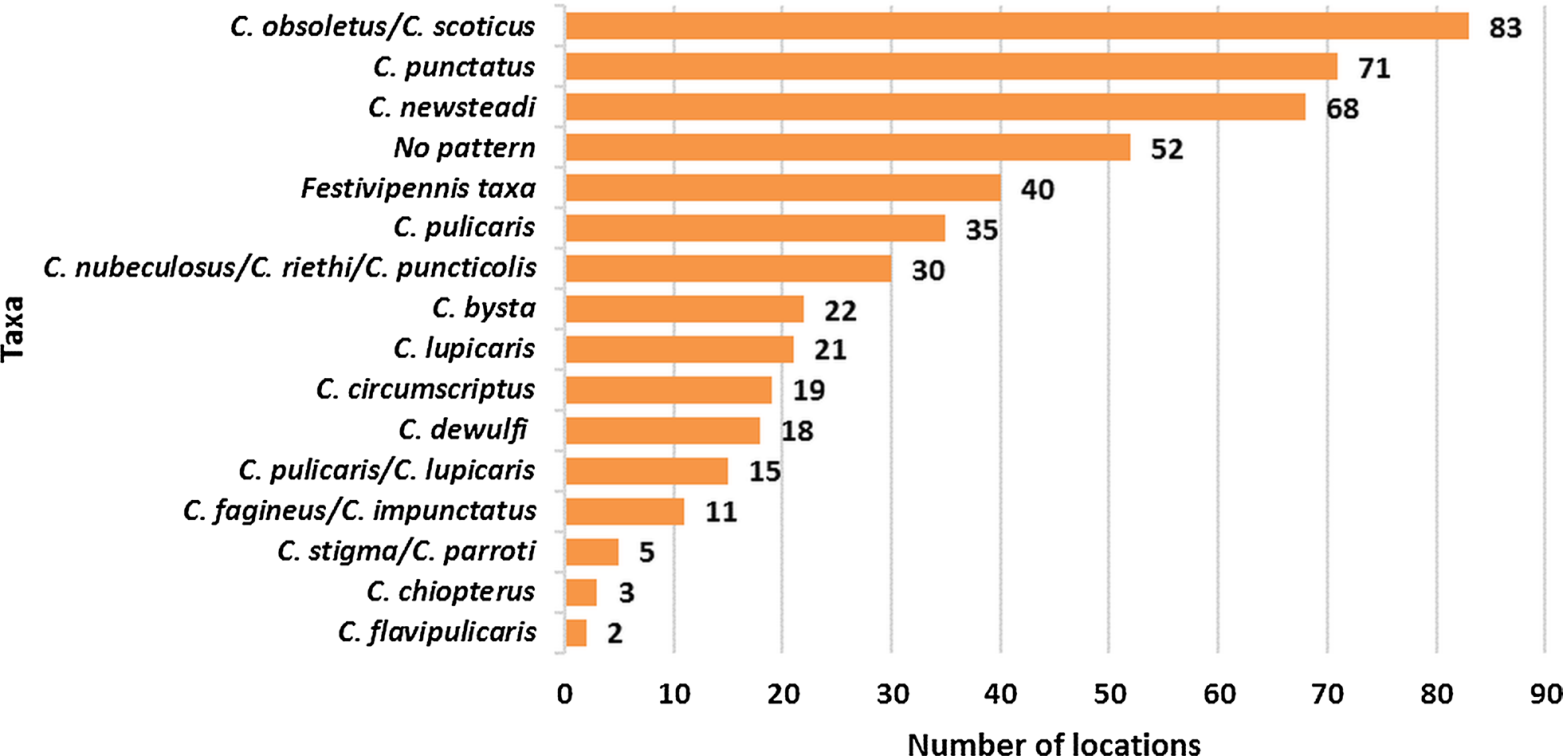
Spatial distribution of *Culicoides* species based on the number of trapping locations in which each species was recorded

The occurrence of *Culicoides* spp. (Fig. 2) showed that the species of Obsoletus complex were found in most locations, followed closely by the two most abundant species of the subgenus *Culicoides*: *C. punctatus* and *C. newsteadi*. Analysis of unique trapping locations at the subgenus level revealed that subgenus *Culicoides* was present in more locations (*n* = 88) than subgenus *Avaritia* (*n* = 83).

**Fig. 3:**
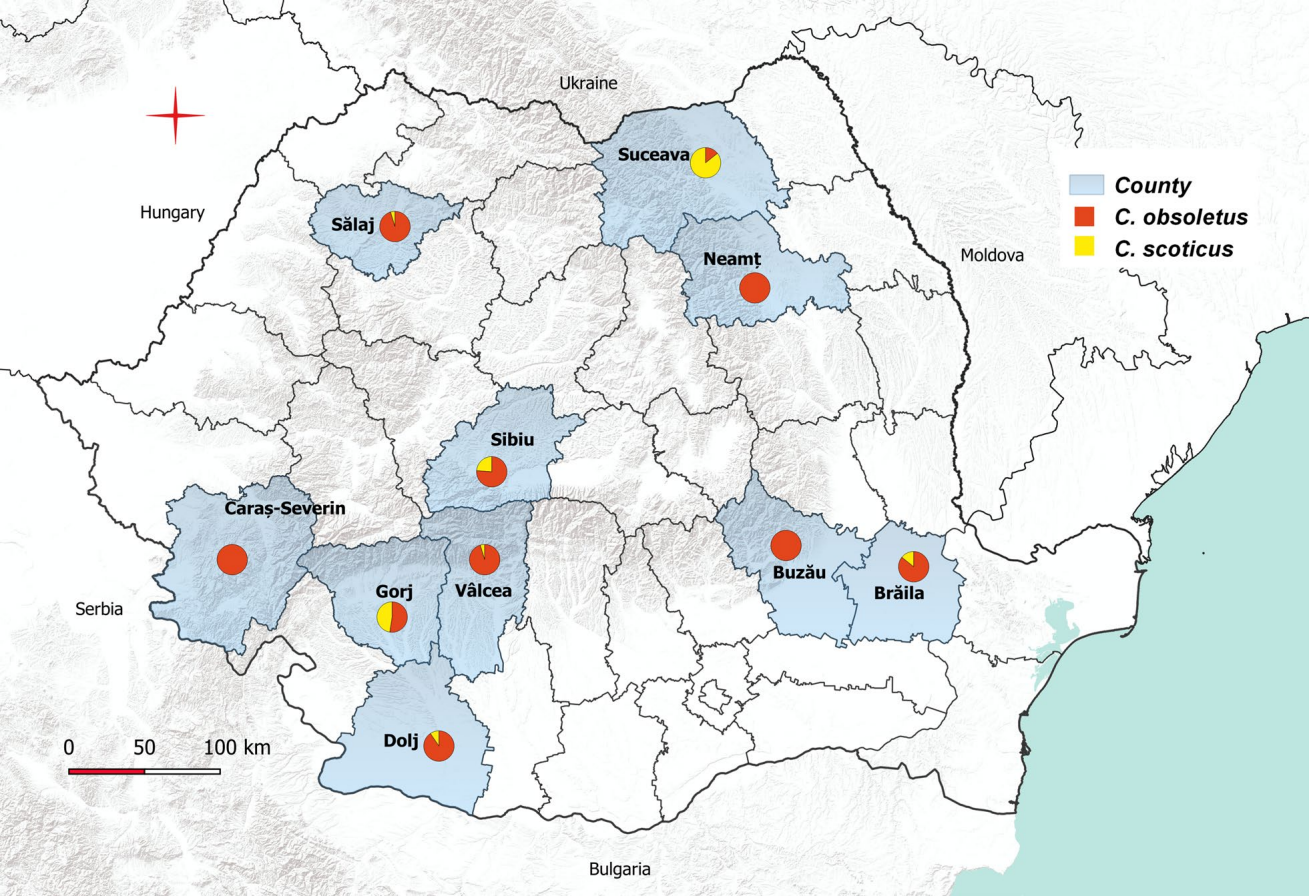
Relative abundance of *C. obsoletus* and *C. scoticus* in 10 counties

The evaluation of the relative abundance of the species of the Obsoletus complex in 10 localities from 10 counties (one locality per county), showed that the most abundant species is *C. obsoletus*, with a percentage range of 14–100% (average of 80.95%). The abundance of *C. scoticus* ranged between 0–85% (average of 19.04%) (Fig. 3).

**Fig. 4:**
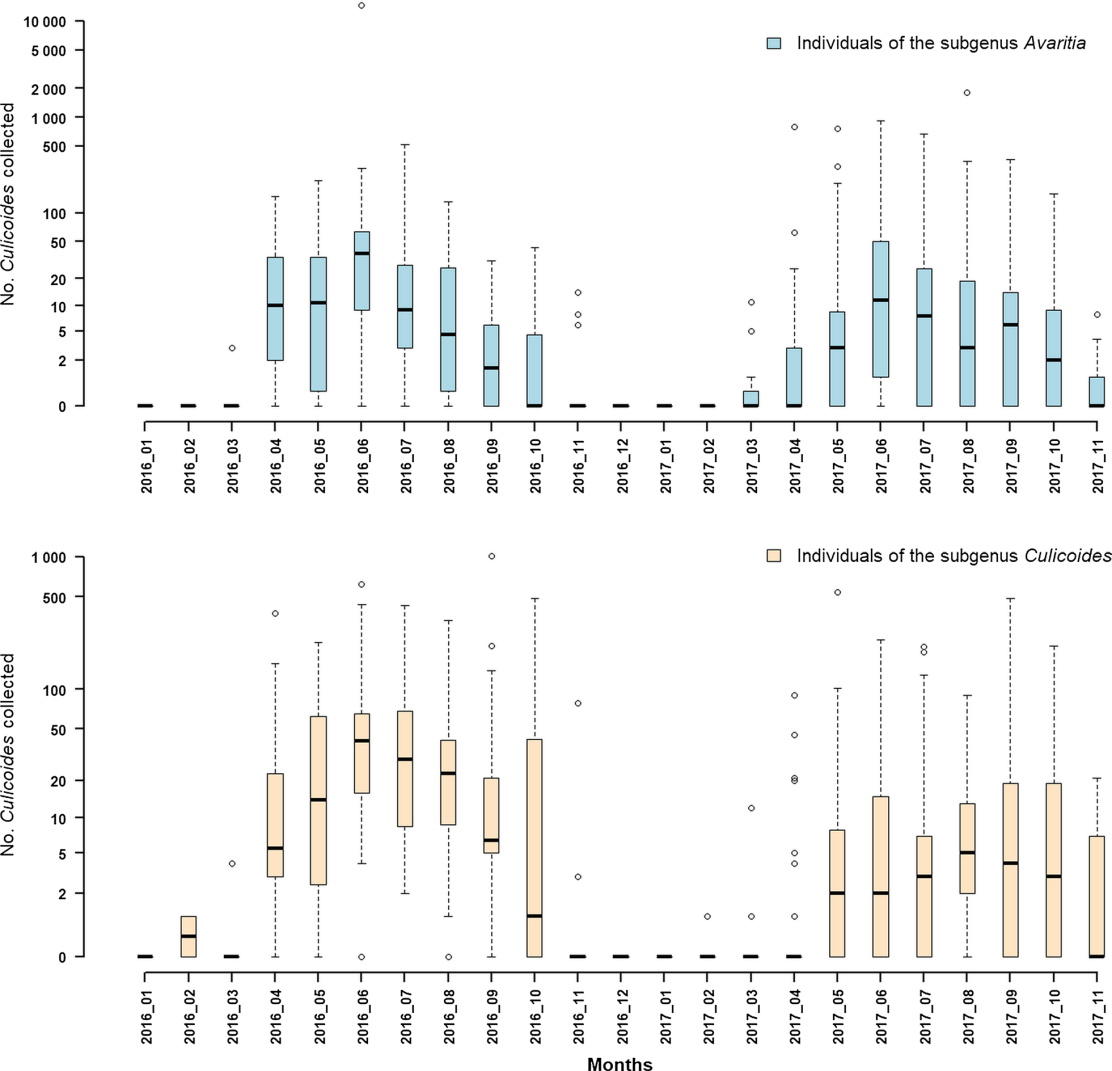
Seasonal abundance of the subgenera *Avaritia* and *Culicoides* in 2016–2017

The seasonal abundance exhibited a peak during early summer (June) and was maintained at lower values during late summer and early-mid autumn. The *Culicoides* spp. activity period started in March and ended in December (Fig. 4).

## Discussion

In Romania, information on the species composition and abundance of the genus *Culicoides* is limited. Georgescu [10] provided the most detailed inventory of species reported to date, mentioning the record of 46 species belonging to seven subgenera. Tomazatos et al. [15] demonstrated the presence for the first time in Romania of three previously described *Culicoides* species. In the context of the increasing incursions of BTV in the Mediterranean regions of Europe since the beginning of the 2000s, the decision to implement an entomological surveillance programme for *Culicoides* biting midge vectors was adopted in 2003. The goals were to evaluate the presence of subgenera known to be involved in the BTV transmission and in order to determine the start and the end of the vector-free period. Within this programme, the identification of the collected individuals was limited to the three most common subgenera (*Avaritia*, *Culicoides* and *Monoculicoides*). The data gathered within the entomological surveillance programme remained unpublished. In this study, we used the existing entomological surveillance infrastructure and, from 2016 to 2017, we evaluated the species composition and relative abundance of the *Culicoides* midges. The relative abundance of the cryptic species belonging to the Obsoletus complex was investigated, integrating the morphological identification with a molecular approach [19], and a picture of *C. obsoletus* and *C. scoticus* distributions across the country was defined (Fig. 3).

The results obtained, regarding the abundance of the Obsoletus complex, are in agreement with the findings of Sarvašová et al. [22] in Slovakia, Goffredo et al. [19] in central Italy and Larska et al. [23] in Poland, who conclude that the species dominating the catches from the Palaearctic area were the morphologically similar *C. obsoletus* and *C. scoticus*.

**Fig. 5:**
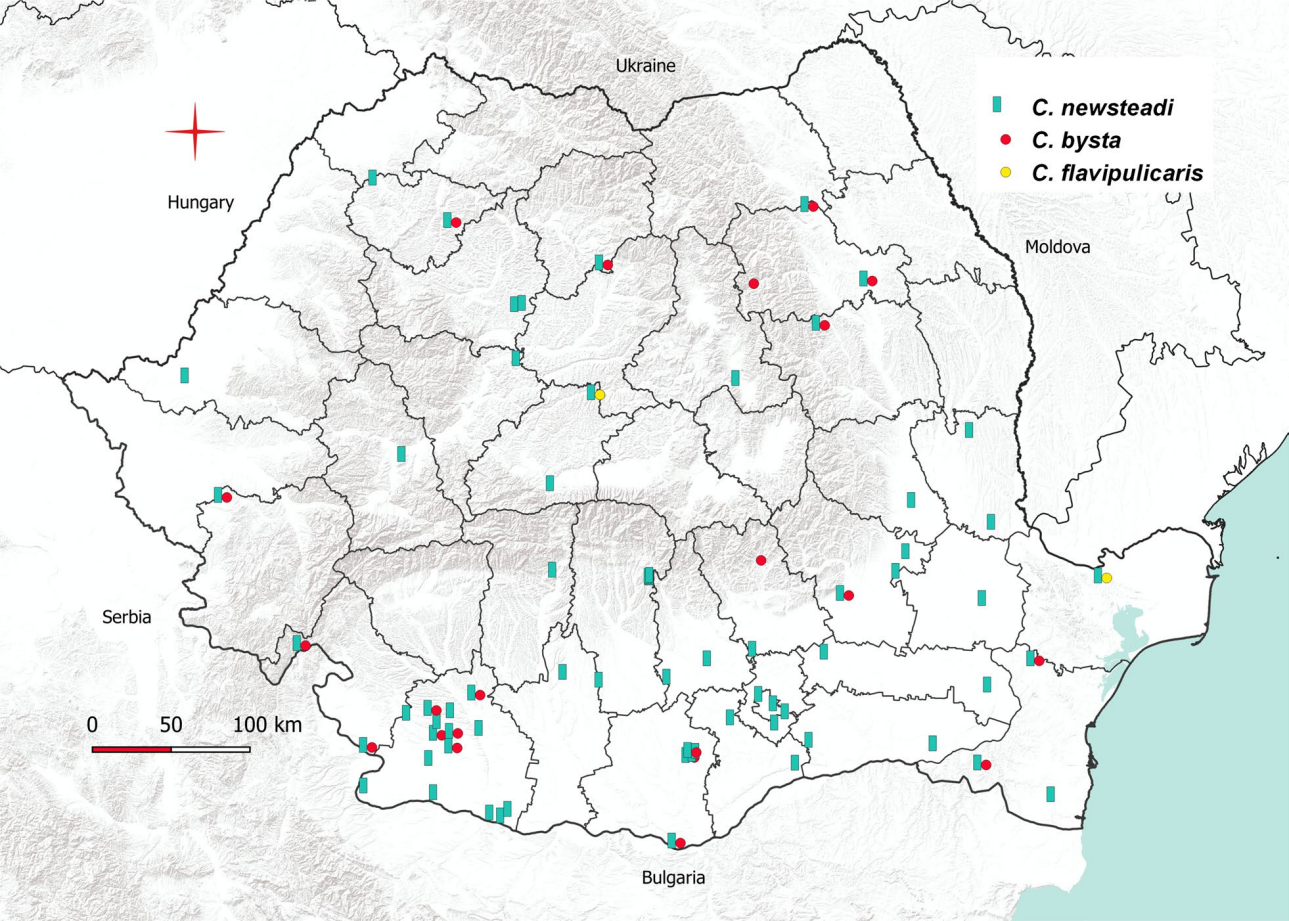
Geographical distribution of *C. newsteadi*, *C. bysta* and C. *flavipulicaris* in Romania (2016–2017)

We recorded for the first time in Romania the species *C. newsteadi*, *C. bysta* and *C. flavipulicaris* (Fig. 5). Considering the publications of Georgescu [10] and Tomazatos et al. [15], we updated the *Culicoides* checklist from Romania to 51 species (Table 2).

**Table 2:** Checklist of the *Culicoides* species described to date in Romania

*Culicoides* spp.	Georgescu [10]	Tomazatos et al. [15]	Present study
*Culicoides* (A*varitia*) *abchazicus* Dzhafarov	×		
*Culicoides* (A*varitia*) *chiopterus* (Meigen)	×		×
*Culicoides* (A*varitia*) *dewulfi* Goetghebuer	×		×
*Culicoides* (A*varitia*) *obsoletus* (Meigen)	×		×
*Culicoides* (A*varitia*) *scoticus* Downes & Kettle	×		×
*Culicoides* (*Beltranmyia*) *circumscriptus* Kieffer	×		×
*Culicoides* (*Beltranmyia*) *manchuriensis* Tokunaga	×		
*Culicodes* (*Beltranmyia*) *salinarius* Kieffer	×		
*Culicoides* (*Beltranmyia*) *sphagnumensis* Williams	×		
*Culicoides* (*Culicoides*) *brunnicans* Edwards	×		
*Culicoides* (*Culicoides*) *bysta* Sarvašová, Kočisová, Candolfi & Mathieu			×
*Culicoides* (*Culicoides*) *delta* Edwards	×		
*Culicoides* (*Culicoides*) *fagineus* Edwards	×		×
*Culicoides* (*Culicoides*) *flavipulicaris* Dzhafarov			×
*Culicoides* (*Culicoides*) *furcillatus* Callot, Kremer & Paradis	×		
*Culicoides* (*Culicoides*) *griseidorsum* Kieffer		×	
*Culicoides* (*Culicoides*) *grisescens* Edwards	×		
*Culicoides* (*Culicoides*)*impunctatus* Goetghebuer	×		×
*Culicoides* (*Culicoides*) *lupicaris* Downes & Kettle	×		×
*Culicoides* (*Culicoides*) *newsteadi* Austen			×
*Culicoides* (*Culicoides*) *parroti* Kieffer	×		×
*Culicoides* (*Culicoides*) *punctatus* (Meigen)	×	×	×
*Culicoides* (*Culicoides*) *pulicaris* (Linnaeus)	×		×
*Culicoides* (*Culicoides*) *simulator* Edwards	×		
*Culicoides* (*Monoculicoides*) *nubeculosus* (Meigen)	×		×
*Culicoides* (*Monoculicoides*) *puncticollis* (Becker)		×	×
*Culicoides* (*Monoculicoides*) *riethi* Kieffer	×	×	×
*Culicoides* (*Monoculicoides*) *stigma* (Meigen)	×		×
*Culicoides* (*Oecacta*) *vexans* (Staeger)	×		
*Culicoides* (*Pontoculicoides*) *saevus* Kieffer	×		
*Culicoides* (*Pontoculicoides*) *tauricus* Gutsevich	×		
*Culicoides* (*Sensiculicoides*) *alazanicus* Dzhafarov	×		
*Culicoides* (*Sensiculicoides*) *comosioculatus* Tokunaga	×		
*Culicoides* (*Sensiculicoides*) *festivipennis* Kieffer	×		×
*Culicoides* (*Sensiculicoides*) *heliophilus* Edwards	×		
*Culicoides* (*Sensiculicoides*) *kibunensis* Tokunaga	×	×	
*Culicoides* (*Sensiculicoides*) *maritimus* Kieffer		×	
*Culicoides* (*Sensiculicoides*) *pictipennis* (Staeger)	×		
*Culicoides* (*Sensiculicoides*) *poperinghensis* Goetghebuer	×		
*Culicoides* (*Sensiculicoides*) *pseudopallidus* Khalaf	×		
*Culicoides* (*Silvaticulicoides*) *achrayi* Kettle & Lawson	×		
*Culicoides* (*Silvaticulicoides*) *duddingstoni* Kettle & Lawson	×		
*Culicoides* (*Silvaticulicoides*) *fascipennis* (Staeger)	×		
*Culicoides* (*Silvaticulicoides*) *pallidicornis* Kieffer	×	×	
*Culicoides* (*Silvaticulicoides*) *picturatus* Kremer & Deduit	×		
*Culicoides* (*Silvaticulicoides*) *subfasciipennis* Kieffer	×	×	
*Culicoides* (*Trithecoides*) *humeralis* Okada	×		
*Culicoides* (*Wirthomyia*) *cameroni* Campbell & Pelham-Clinton	×		
*Culicoides* (*Wirthomyia*) *segnis* Campbell & Pelham-Clinton	×		
*Culicoides pumilus* (Winnertz)	×		
*Culicoides riouxi* Callot & Kremer	×		

The epidemiological role of at least 10 *Culicoides* species described in our study, *C. obsoletus*, *C. scoticus*, *C. dewulfi*, *C. chiopterus*, *C. punctatus*, *C. newsteadi*, *C. pulicaris*, *C. lupicaris*, *C. nubeculosus* and *C. circumscriptus*, was previously demonstrated or assumed following research activities of numerous authors. Goffredo et al. [24] described the involvement of the species of the Obsoletus complex and *C. newsteadi* in the transmission of BTV-4. Furthermore, the same authors, as well as Foxi et al. [25], indicated species of Obsoletus complex as confirmed vectors and *C. newsteadi*, *C. dewulfi*, *C. pulicaris* and *C. punctatus* as probable vectors capable of transmitting the BTV-1. De Liberato et al. [26] managed to isolate BTV2 from the Obsoletus complex. In Germany, Clausen et al. [27] reported positive results on real time RT-PCR testing for BTV-8 on pools of *C. obsoletus* and *C. pulicaris*. BTV was detected by real time RT-PCR on pools of *C. chiopterus* captured in the Netherlands [28]. The competence for transmission of the SBV was suggested for the species of the Obsoletus complex [29–31], but also for *C. chiopterus*, *C. dewulfi*, *C. pulicaris*, *C. newsteadi*, *C. lupicaris* and *C. nubeculosus* [32, 33]. The involvement of *Culicoides* spp. in the transmission of the AHS virus was demonstrated for *C. imicola* [34, 35]. However, studies on *Culicoides* specimens caught during the AHS epizootic in Spain (1988) have shown that the virus can also be isolated from pools composed exclusively of *C. obsoletus*, *C. pulicaris*, *C. odiatus* and *C. cataneii* [36]. In relation to the above, this study shows that in Romania the most important vector species for BTV and SBV are commonly present and abundant.

The vector population composition and abundance was similar to those of other countries at the same latitude of Romania, where different BTV serotypes occurred in the last decade. Similarly to Romania, the subgenera *Avaritia* and *Culicoides* form the bulk of the species list in Balkans and western European countries. What differentiates the results of the entomological surveillance activities is the proportion of the two subgenera in different European regions. Pudar et al. [37] reported that the most frequent species in Bosnia and Herzegovina, Bulgaria, Croatia belong to the subgenus *Culicoides* (*C. punctatus*, 66% of sites; *C. newsteadi*, 57% of sites; and *C. pulicaris*, 30% of sites) followed by the subgenus *Avaritia* (*C. obsoletus/C. scoticus*, 30% of sites). In Hungary, following the implementation of the official entomological surveillance programme for BT, it was concluded that the subgenus *Culicoides* was the most abundant (63.5% of the captured midges) followed by the subgenera *Monoculicoides* (12.3%) and *Avaritia* (11.7%) [38].

The presence of *C. dewulfi* and *C. chiopterus* (larvae of these species are known to develop in animal dungs [39, 40]), indicates that favorable breeding sites could closely follow the presence of domestic and wild animal feeding hosts [41].

Determining species composition and assessing abundance of the main vector species is essential to produce accurate abundance and distribution maps, and then maps of transmission risk for *Culicoides*-borne diseases, that can be used to focus surveillance and control programmes.

## Conclusions

Studies on the species composition and abundance of the genus *Culicoides* in Romania are necessary, especially in the context of BT evolution at a national level, since 2014. Our research confirmed the existence of known vector species involved in the transmission of important animal viruses, such as BTV and SBV but also provided an update of known *Culicoides* species in Romania. We consider that the species composition and abundance of insects of the genus *Culicodes* in Romania represent not only a permanent risk factor in relation to incursion and establishment at a national level of other BTV serotypes, but also a possible favoring factor for establishment, under certain conditions, of other exotic vector-borne diseases such as African horse sickness and epizootic haemorrhagic disease.

## Data Availability

All data generated or analysed during this study are included in this published article.

## Abbreviations

PCR: polymerase chain reaction
ITS: internal transcribed spacer
BT: bluetongue
BTV: bluetongue virus
SBV: Schmallenberg virus
AHS: African horse sickness

## References

[1] Mellor PS, Boorman J, Baylis M (2000). *Culicoides* biting midges: their role as arbovirus vectors. Annu Rev Entomol..

[2] Tabachnick WJ (2004). *Culicoides* and the global epidemiology of bluetongue virus infection. Vet Ital..

[3] Rasmussen DL, Kristensen B, Kirkeby C, Belsham JG (2012). Culicoids as vectors of Schmallenberg virus. Emerg Infect Dis..

[4] Zientara S, Weyer CT, Lecollinet S (2015). African horse sickness. Rev Sci Tech..

[5] Rushton J, Lyons N (2015). Economic impact of bluetongue: a review of the effects on production. Vet Ital..

[6] Tilibașa EM, Popescu D, Badea C, Hora FȘ, Dărăbuș G (2014). A report regarding first occurrence of bluetongue in Romania. Sci Works Series C Vet Med.

[7] Gonciarov M, Coman C (2014). Risk factors, incidence and prevalence of bluetongue in Romania and worldwide in the last decade. Sci Works Series C Vet Med.

[8] Diaconu C, Hristescu D, Bărbuceanu F, Popovici A, Tamba P, Moțiu R (2017). Morphoclinical aspects and diagnosis regarding bluetongue outbreaks in Romania. Rev Rom Med Vet..

[9] Moț D, Nichita I, Tîrziu E, Moț T (2018). Bluetongue in Europe and Romania in the last years. J Anim Sci Biotechno..

[10] Georgescu DA (2000). Fauna României, Insecta, Volumul XI, Fascicula 14, Diptera, Familia Ceratopogonidae, Genul *Culicoides*.

[11] Dascălu L, Ionescu A, Rizac V (2007). Weather stations and the data reports from Romania as part of the East-BTNet project. Vet Ital..

[12] Oprescu I, Dărăbuș G, Morariu S, Mederle N, Ilie M, Panici Z (2008). The dynamics of *Culicoides* insect populations in didactical and experimental station Timișoara, between May and September 2005. Lucrări științifice de medicină veterinară..

[13] Ilie A, Șerban C, Imre M, Sorescu D, Ilie M, Imre K (2013). A survey on *Culicoides* (Diptera: Ceratopogonidae) in Gorj county, Romania. Lucrări științifice medicină veterinară..

[14] Tilibașa EM, Badea C, Hora FȘ, Dărăbuș G. A study on dynamics and prevalence in May–June 2013 of *Culicoides* spp., in Timiș county. Lucrări științifice de medicină veterinară 2014;47:112–9.

[15] Tomazatos A, Jӧst H, Schulze J, Spînu M, Schmidt CJ, Cadar D (2020). Blood-meal analysis of *Culicoides* (Diptera: Ceratopogonidae) reveals a broad host range and new species records for Romania. Parasit Vectors..

[16] Campbell J, Pelham-Clinton E (1960). A taxonomic review of the British species of “*Culicoides*” Latreille (Diptera, Ceratopogonidae). Proc R Soc Edinb B Biology..

[17] Delécolle JC. Nouvelle contribution a l’etude systématique et iconographique des espéces du genre *Culicoides* (Diptera: Ceratopogonidae) du Nord-Est de la France. These d’Universite. Universite Louis Pasteur de Strasbourg UER Sciences Vie et Terre, France; 1985.

[18] Goffredo M, Meiswinkel R (2004). Entomological surveillance of bluetongue in Italy: methods of capture, catch analysis and identification of *Culicoides* biting midges. Vet Ital..

[19] Goffredo M, Meiswinkel R, Federici V, Di Nicola F, Manicini G, Ippoliti C (2016). The ‘*Culicoides obsoletus* group’ in Italy: relative abundance, geographic range, and role as vector for bluetongue virus. Vet Ital..

[20] Gomulski LM, Meiswinkel R, Delecolle JC, Goffredo M, Gasperi G (2005). Phylogenetic relationships of the subgenus *Avaritia* Fox, 1955 including *Culicoides obsoletus* (Diptera, Ceratopogonidae) in Italy based on internal transcribed spacer 2 ribosomal DNA sequences. Syst Entomol..

[21] Matthieu B, Perrin A, Baldet T, Delecolle JC, Albina E, Cetre-Sossah C (2007). Molecular identification of western European species of the Obsoletus complex (Diptera: Ceratopogonidae) by internal transcribed spacer-1 rDNA multiplex polymerase chain reaction assay. J Med Entomol..

[22] Sarvašová A, Goffredo M, Sopoliga I, Savini G, Kocisova A (2014). *Culicoides* midges (Diptera: Ceratopogonidae) as vectors of orbiviruses in Slovakia. Vet Ital..

[23] Larska M, Grochowska M, Lechowski L, Zmudzinski JF. Abundance and species composition of *Culicoides* spp. biting midges near cattle and horse in south-eastern Poland. Acta Parasitol. 2017;62:739–47. 10.1515/ap-2017-0089 29035852

[24] Goffredo M, Catalani M, Federici V, Portanti O, Marini V, Mancini G (2015). Vector species of *Culicoides* midges implicated in the 2012–2014 bluetongue epidemics in Italy. Vet Ital..

[25] Foxi C, Delrio G, Falchi G, Marche GM, Satta G, Ruiu L (2016). Role of different *Culicoides* vectors (Diptera: Ceratopogonidae) in bluetongue virus transmission and overwintering in Sardinia (Italy). Parasit Vectors..

[26] De Liberato C, Scavia G, Lorenzetti R, Scaramozzino P, Amaddeo D, Cardeti G (2005). Identification of *Culicoides obsoletus* (Diptera: Ceratopogonidae) as a vector of bluetongue virus in central Italy. Vet Rec..

[27] Clausen PH, Stephan A, Bartsch S, Jandowski A, Hoffmann-KP, Schein E, et al. Seasonal dynamics of biting midges (Diptera: Ceratopogonidae, *Culicoides* spp.) on dairy farms of central Germany during the 2007/2008 epidemic of bluetongue. Parasitol Res. 2009;105:381–6. 10.1007/s00436-009-1417-x 19333620

[28] Dijkstra E, van der Ven IJ, Hӧlzel DR, Van Rijn PA, Meiswinkel R (2008). *Culicoides chiopterus* as a potential vector of bluetongue virus in Europe. Vet Rec..

[29] Barber J, Lara EH, Silk R, Veronesi E, Gubbins S, Bankowska BK (2018). Blood-feeding, susceptibility to infection with Schmallenberg virus and phylogenetics of *Culicoides* (Diptera: Ceratopogonidae) from the United Kingdom. Parasit Vectors..

[30] Pages N, Talavera S, Verdun M, Pujol N, Valle M, Bensaid A (2018). Schmallenberg virus detection in *Culicoides* biting midges in Spain: first laboratory evidence for highly efficient infection of *Culicoides* of the Obsoletus complex and *Culicoides imicola*. Transbound Emerg Dis..

[31] Goffredo M, Monaco F, Capelli G, Quaglia M, Federici V, Catalani M (2013). Schmallenberg virus in Italy: a retrospective survey in *Culicoides* stored during the bluetongue Italian surveillance program. Prev Vet Med..

[32] De Regge N, Deblauwe I, De Deken R, Vantieghem P, Madder M, Geysen D, et al. Detection of Schmallenberg virus in different *Culicoides* spp. by real-time RT-PCR. Transbound Emerg Dis. 2012;59:471–5. 10.1111/tbed.12000 23025501

[33] Segard A, Gardes L, Jacquier E, Grillet C, Mathieu B, Rakotoarivony I (2018). Schmallenberg virus in *Culicoides* Latreille (Diptera: Ceratopogonidae) populations in France during 2011–2012 outbreak. Transbound Emerg Dis..

[34] Carpenter S, Mellor SP, Assane GF, Garros C, Venter JG (2017). African horse sickness virus: history, transmission, and current status. Annu Rev Entomol..

[35] Bourquia M, Garros C, Rakotoarivony I, Gardes L, Huber K, Boukhari I (2019). Update of the species checklist of *Culicoides* Latreille, 1809 biting midges (Diptera: Ceratopogonidae) of Morocco. Parasit Vectors..

[36] Mellor PS, Boned J, Hamblin C, Graham S (1990). Isolations of African horse sickness virus from vector insects made during the 1988 epizootic in Spain. Epidemiol Infect..

[37] Pudar D, Petric D, Allene X, Alten B, Ayhan N, Cvetkovikj A (2018). An update of the *Culicoides* (Diptera: Ceratopogonidae) checklist for the Balkans. Parasit Vectors..

[38] Szell Z, Bodrogi B, Sreter T. *Culicoides* - species: lesser-known vectors of emerging diseases. Review and results of the national monitoring program. Magy Allatorvosok Lapja. 2016;138:361–72.

[39] Zimmer JY, Haubruge E, Francis F, Bortels J, Simonon G, Losson B (2008). Breeding sites of bluetongue vectors in northern Europe. Vet Rec..

[40] Steinke S, Lühken R, Kiel E (2015). Impact of freezing on the emergence of *Culicoides chiopterus* and *Culicoides dewulfi* from bovine dung. Vet Parasitol..

[41] Lühken R, Kiel E, Steinke S, Fladung R (2015). Topsoil conditions correlate with the emergence rates of *Culicoides chiopterus* and *Culicoides dewulfi* (Diptera: Ceratopogonidae) from cowpats. Parasitol Res..

